# 

**Published:** 1881-04

**Authors:** 


### CONTENTS.

### COMMUNICATIONS.

Alveolar Abscess........................................................133
Literary Recreation.....................................................137
Self-Confidence an Essential Element in the Character of the Ideal or Model Dentist........................................................160

### DISCUSSIONS.

Thirty-Seventh Annual Meeting of the Mississippi Valley Dental Society........................................................142

### PROCEEDINGS.

Mississippi Valley Association of Dental Surgeries—Thirty-Seventh Annual Meeting........................................................154

### SELECTIONS.

Absorbent Cotton........................................................166

### CORRESPONDENCE.

Hospital Reports........................................................168
Dentistry in the Northwest........................................................169

### EDITORIAL.

Ohio College of Dental Surgery........................................................171
Commencement........................................................173
Association........................................................174
A Proper Recognition........................................................176
Annual Meeting........................................................176

